# Brazilian version of the Cognitive Risk Profile for Pain Scale: translation, cross-cultural adaptation, and validation

**DOI:** 10.1590/1516-3180.2025.0007.R1.23092025

**Published:** 2026-02-13

**Authors:** Lucianna Mirelle de Sá Trabulsi, Almir Vieira Dibai, André Pontes-Silva, Sulamizia Filomena Costa de Jesus, Letícia Padilha Mendes, Gabriel Gardhel Costa Araujo, Cid André Fidelis-de-Paula-Gomes, Daniela Bassi-Dibai, Adriana Sousa Rêgo

**Affiliations:** I RN, Programa de Pós-Graduação em Gestão de Programas e Serviços de Saúde, Universidade Ceuma, São Luís (MA), Brazil.; II PT; Professor, Programa de Pós-Graduação em Educação Física, Universidade Federal do Maranhão (UFMA), São Luís (MA), Brazil.; III BPE; Researcher, Programa de Pós-Graduação em Fisioterapia, Universidade Federal de São Carlos (UFSCar), São Carlos (SP), Brazil.; IV PT; Programa de Pós-Graduação em Educação Física, Universidade Federal do Maranhão (UFMA), São Luís (MA), Brazil.; V Researcher; Programa de Pós-Graduação em Educação Física, Universidade Federal do Maranhão (UFMA), São Luís (MA), Brazil.; VI PT; Programa de Pós-Graduação em Educação Física, Universidade Federal do Maranhão (UFMA), São Luís (MA), Brazil.; VII PT; Professor, Programa de Pós-Graduação em Ciências da Reabilitação, Universidade Nove de Julho (Uninove), São Paulo (SP), Brazil.; VIII PT; Professor, Programa de Pós-Graduação em Gestão de Programas e Serviços de Saúde, Universidade Ceuma, São Luís (MA), Brazil.; IX Professor, Programa de Pós-Graduação em Gestão de Programas e Serviços de Saúde, Universidade Ceuma, São Luís (MA), Brazil.

**Keywords:** Chronic pain, Musculoskeletal system, Measurement properties, Cognitive behavioral therapy, Pain, COSMIN, PROMs, Cognitive Dysfunction

## Abstract

**OBJECTIVES:**

To translate, cross-culturally adapt, and validate the Cognitive Risk Profile for Pain (CRPP) scale for Brazilians with chronic pain.

**METHODS:**

We conducted a questionnaire-based validation study. Patients (males and females) with pain in any part of the body for > 3 months were included (n = 191). The participants were assessed using the CRPP scale, the Numeric Pain Rating Scale (NPRS), the Hospital Anxiety and Depression Scale (HADS), the Pain-Related Catastrophizing Thoughts Scale (PCTS), and the Brunel Mood Scale (BRUMS). After the translation, cross-cultural adaptation, and validation of the CRPP scale, we tested for ceiling or floor effects, construct validity, reliability, and internal consistency. Finally, the second application of the CRPP scale was used to measure test-retest reliability.

**RESULTS:**

Most participants were female, over 36 years of age, overweight, physically active, and had an average pain history of approximately 41 months. No ceiling or floor effects are observed. Nine domains of the CRPP scale correlated with the NPRS score, two domains of the HADS and PCTS, and six domains of the BRUMS. All CRPP domains demonstrated adequate reliability and internal consistency.

**CONCLUSION:**

The Brazilian version of the CRPP scale demonstrated adequate measurement properties in patients with chronic pain.

## INTRODUCTION

 Chronic pain affects approximately 116 million adults and costs approximately $635 billion annually in medical treatment and lost worker productivity.^1^ It is a major public health problem. The prevalence of chronic pain is continuously investigated.^2^ The multidimensional aspect of pain and the difficulties in adequately measuring and characterizing pain are recognized. Instruments that allow the accurate and consistent measurement of chronic pain are warranted. The cognitive risk profile for pain, as reported by the patient, is also necessary.^3^

 Although assessment of the cognitive risk profile for pain is important in assessment routines, it is complex and challenging to elucidate in patients with chronic pain. The constructs included in the cognitive risk profile of pain are difficult to measure. For example, the philosophical beliefs about pain, denial that mood affects pain, denial that pain affects mood, perception of blame, inadequate support, disability entitlement, desire for medical breakthrough, skepticism of a multidisciplinary approach, and conviction of hopelessness.^4^

 Cook and DeGood^4^ developed the Cognitive Risk Profile for Pain Scale (CRPP), which showed adequate internal consistency and construct validity in an American population. To date, the CRPP scale has not been adapted for the Brazilian population according to international recommendations^5-7^ preventing its use in research and routine clinical assessments. Therefore, this study aimed to translate, cross-culturally adapt, and validate the CRPP scale for Brazilian patients with chronic pain. 

## METHODS

### Study design and ethical aspects

 A questionnaire validation study was conducted according to the guidelines for the process of cross-cultural adaptation of self-report measures^3^ and the Consensus-Based Standards for the Selection of Health Measurement Instruments (COSMIN).^6^

 Data collection for this study was conducted online using the free Google Forms platform (Mountain View, California) and disseminated through social networks and physical therapy clinics. This study was approved by the Research Ethics Committee of Universidade Ceuma (report no. 4.555.379). 

### Translation and cross-cultural adaptation

 Permission to adapt the questionnaire into Brazilian Portuguese was granted by email from one of the authors (Dr. Andrew J. Cook).^4^ The process of translation and cross-cultural adaptation of the CRPP scale into Brazilian Portuguese followed the recommendations of Beaton et al.^3^ and was developed in five phases: translation and cross-cultural adaptation, synthesis of translations, back-translation of the questionnaire, analysis, and testing of the final version of the CRPP scale cross-culturally adapted into Brazilian Portuguese. 

 Translation: Two independent translators (a physiotherapist with 10 years of experience in the field and an English teacher with 20 years of experience in translation without technical knowledge in the health field), native speakers of Brazilian Portuguese fluent in English, translated the original version of the CRPP scale into Brazilian Portuguese. 

 Synthesis of translations: After discussions and revisions, two translators, under the supervision of one of the researchers, independently synthesized two versions of the translated questionnaire, which were discussed to produce a single version in a consensual manner. 

 Back-translation: Two independent translators (with no technical knowledge of health issues) and native and fluent Portuguese speakers translated the Portuguese version of the CRPP scale back into English without prior knowledge of the original version of the questionnaire. 

 Analysis by a committee of experts: Four specialists in the field of rehabilitation, together with the four translators involved in the project, reviewed all translated and back-translated versions to correct any discrepancies and arrived at the pre-final version of the CRPP scale in a form agreed upon by all members of the committee. 

 Pre-final version test: The pre-final version of the CRPP scale was administered to 30 individuals with chronic pain whose first language was Brazilian Portuguese. Participants read and completed the questionnaire, and at the end of completion, they indicated their understanding of the pre-final version of the CRPP scale by checking a box with "Yes" or "No" answers for each item in the questionnaire. If the items were not understood by more than 20% of the participants, they were reformulated and retested with a new sample of 30 participants until the desired level of understanding was reached. 

### Sampling and participants

 The sample size was calculated according to the COSMIN recommendation of at least 100 participants.^7^ The following inclusion criteria were considered: patients of both sexes; age 18 years or older; pain for more than three months in any part of the body; participants receiving treatment for pain, whether medical, pharmacological, physiotherapeutic or psychotherapeutic; literate. The exclusion criteria were as follows: diagnosis of severe cognitive or psychiatric illness, and non-Brazilian participants. 

 After defining the final version of the CRPP scale, the following measurement properties were evaluated: construct validity and test-retest reliability. This was accomplished by administering CRPP twice, one week apart. First, the CRPP scale and the following instruments were used to validate the construct: Numeric Pain Rating Scale (NPRS),^8^ Hospital Anxiety and Depression Scale (HADS),^9^ Pain-Related Catastrophizing Thoughts Scale (PCTS),^10^ Brunel Mood Scale (BRUMS).^11^ In the second application, the CRPP scale was administered to measure test-retest reliability. 

### Cognitive Risk Profile for Pain scale (CRPP)

 The CRPP scale is a self-report instrument designed to aid clinical risk assessment and treatment planning for patients with chronic pain. It consists of 53 items with 6 response options: 1 – I strongly agree; 2 – I somewhat agree; 3 – I somewhat agree; 4 – I somewhat disagree; 5 – I partially disagree; 6 – I strongly disagree. However, several items have reverse scores (1 = 6; 2 = 5; 3 = 4; 4 = 3; 5 = 2; 6 = 1), namely: items 2–5, 7, 11–14, 16–18, 23, 24, 26, 28–30, 32, 35–43, 45–48, 51–53. 

 CRPP has nine domains: philosophic beliefs about pain (items 16, 17, 29, 30, 35, and 45); denial that mood affects pain (items 8, 19, 25, 31, and 53); denial that pain affects mood (items 10, 21, 33, 34, 49, and 50); perception of blame (items 9, 12, 23, 24, and 48); inadequate support (items 6, 11, 37, 39, 41, and 43); disability entitlement (items 13, 26, 32, 42, 47, and 51); desire for medical breakthrough (items 2, 5, 28, 36, 40, 46, and 52); skepticism of multidisciplinary approach (items 1, 15, 20, 22, 27, and 44); conviction of hopelessness (items 3, 4, 7, 14, 18, and 38). 

 For each domain, the average of the responses was calculated, generating scores ranging from one to six. High values indicate greater cognitive risk.^4^ The Brazilian version of the CRPP scale is available at https://questionariosbrasil.blogspot.com/. 

### Others scales

 NPRS measures pain intensity using a series of 11 numbers (from 0 to 10), with 0 representing "no pain" and 10 representing "maximum imaginable pain." This instrument was validated in Portuguese.^8^

 The HADS measures symptoms of anxiety and depression using 14 items (seven items for anxiety and seven items for depression). To calculate a score for each domain, the answered items were added together, resulting in a score varying from 0 to 21. Higher scores indicated more severe symptoms. This instrument was previously validated in Brazil.^9^

 The PCTS measures catastrophic thinking with 9 items. To calculate the total score, all items were added and divided by the number of items answered, resulting in a score varying from 0 to 5. Higher scores indicated greater catastrophizing. This instrument was previously validated in Brazil.^10^

 The BRUMS assesses mood using 24 items grouped into six domains: anger, confusion, depression, fatigue, tension, and vigor. Each domain contains four items. The sum of the responses for each subscale yielded a score ranging from 0 to 16. Higher scores indicated a worse mood. This instrument was previously validated in Brazil.^11^

### Statistical analysis

 Descriptive statistics were performed, and the variables are presented as means and standard deviations, or absolute and relative frequencies. SPSS software (version 17.0, Chicago) was used to analyze the descriptive statistics, reliability, internal consistency, and construct validity. 

 Internal consistency was calculated using Cronbach’s alpha to determine whether redundant or heterogeneous items were present in the questionnaire. Cronbach’s alpha > 0.7 was considered adequate.^6^ Reliability was assessed based on a test-retest model using the intraclass correlation coefficient (ICC). An ICC > 0.75 was considered adequate.^12^ In addition, we calculated the standard error of measurement (SEM) and minimum detectable change (MDC).^13^

 For construct validity, the Spearman correlation coefficient (rho) was used to determine the magnitude of the correlation between the CRPP scale and other instruments. Since there is no instrument with a similar construct used in Brazil, our hypothesis is that correlations with instruments measuring related but different constructs should vary between 0.3 and 0.5.^13^ Ceiling and floor effects were evaluated in this study. By definition, these effects occur when more than 15% of the study participants reach the minimum or maximum questionnaire scores.^13^

## RESULTS

 Two adjustments were made to the Brazilian version of the CRPP scale: in item 9, the term "medical care" was changed to "care provided by health team care"; in item 39, the term "insurance providers" was expanded to "insurance providers, health insurance, and/or public health system". The preliminary version of the CRPP scale was administered to 30 patients with chronic pain. Item 12 was understood by 28 participants (93.33%). All other 52 items were understood by 100% of the respondents. 

 The study sample comprised 191 participants. Most participants were female, over 36 years old, overweight, physically active, and did not use tobacco or alcohol. The most common pain locations were the lumbar spine, cervical spine, knees, and shoulders. The mean duration of pain was slightly longer than 41 months (Table 1). Among the data obtained, the variables anxiety and depression of the HADS and vigor and mental confusion of the BRUMS presented the highest means as well as the highest standard deviations of the entire sample (Table 2). 

**Table 1:** Participant characteristics (n = 191)

**Variables**		**Mean (± SD) or Number (%)**
Sex (female)		128 (67%)
Age (years)		36.56 (12.27)
Body mass (kg)		72.16 (15.63)
Stature (m)		1.64 (0.09)
Body mass index (kg/m²)		26.40 (4.81)
Marital status		
	Single	104 (54.5%)
	Married	72 (37.7%)
	Divorced	13 (6.8%)
	Widowed	2 (1%)
Education level		
	Incomplete primary education	5 (2.6%)
	Complete primary education	6 (3.1%)
	Incomplete secondary education	5 (2.6%)
	Complete secondary education	20 (10.5%)
	Incomplete undergraduate degree	42 (22%)
	Complete undergraduate degree	36 (18.8%)
	Incomplete post-graduation	19 (9.9%)
	Complete post-graduation	58 (30.5%)
Physical activity (yes)		138 (72.3%)
Smoker		
	Yes	2 (1%)
	No	186 (97.4%)
	Ex-smoker	3 (1.6%)
Alcoholism		
	No	90 (47.1%)
	Rarely	75 (39.3%)
	Once/week	24 (12.6%)
	Daily	2 (1%)
Pain time (months)		41.72 (62.36)
Main body pain sites		
	Low back	90 (47.1%)
	Neck	26 (13.6%)
	Knee	22 (11.5%)
	Shoulder	18 (9.4%)
	Head	7 (3.7%)
	Foot	7 (3.7%)
	Hip	5 (2.6%)
	Leg	4 (2.1%)
	Others	12 (6.3%)

**Table 2:** Instrument scores (n = 191)

**Instrument**		**Mean (SD)**
Numeric Pain Rating Scale (score, 0–10)		6.12 (2.31)
Hospital Anxiety and Depression Scale (score, 0-21)		
	Anxiety	9.05 (4.65)
	Depression	6.61 (3.88)
Pain-Related Catastrophizing Thoughts Scale (score, 0–5)		1.78 (1.31)
Brunel Mood Scale (score, 0–16)		
	Tension	5.95 (2.44)
	Depression	5.83 (3.84)
	Anger	5.76 (3.83)
	Vigor	6.52 (2.78)
	Fatigue	4.92 (2.28)
	Mental confusion	6.41 (3.15)
Cognitive Risk Profile for Pain Scale (score, 1–6)		
	D1	4.27 (0.91)
	D2	2.7 (1.06)
	D3	3.02 (1.25)
	D4	2.74 (1.03)
	D5	3.25 (0.97)
	D6	2.48 (1.32)
	D7	4.26 (0.87)
	D8	1.71 (0.6)
	D9	3.14 (1.27)

D1, philosophic beliefs about pain; D2, denial that mood affects pain; D3, denial that pain affects mood; D4, perception of blame; D5, inadequate support; D6, disability entitlement; D7, desire for medical breakthrough; D8, skepticism of multidisciplinary approach; D9, conviction of hopelessness.

 The nine domains of the CRPP scale correlated with the NPRS score, anxiety, and depression domains of the HADS, PCTS, and six domains of the BRUMS. A significant correlation was observed with magnitudes varying from 0.145 to 0.658 (Table 3). 

**Table 3:** Correlation between the domains of the Cognitive Risk Profile for Pain scale (CRPP) and other instruments (n = 191)

**Instruments**		**CRPP**								
		**D1**	**D2**	**D3**	**D4**	**D5**	**D6**	**D7**	**D8**	**D9**
NPRS		0.240*	0.041	0.331*	0.117	0.263*	0.293*	0.055	0.051	0.345*
HADS										
	Anxiety	0.232*	0.074	0.428*	0.308*	0.360*	0.239*	0.211*	0.270*	0.439*
	Depression	0.196*	0.053	0.359*	0.265*	0.342*	0.231*	0.148*	0.274*	0.412*
	PCTS	0.376*	0.003	0.497*	0.304*	0.360*	0.232*	0.317*	0.658*	0.596*
Brunel Mood Scale										
	Tension	0.212*	0.044	0.291*	0.255*	0.296*	0.154*	0.108	0.163*	0.275*
	Depression	0.260*	0.075	0.431*	0.271*	0.369*	0.225*	0.136	0.246*	0.478*
	Anger	0.262*	0.062	0.413*	0.268*	0.321*	0.154*	0.178*	0.260*	0.472*
	Vigor	0.230*	0.153*	0.382*	0.274*	0.331*	0.204*	0.206*	0.145*	0.441*
	Fatigue	0.260*	0.098	0.332*	0.232*	0.320*	0.160*	0.151*	0.087	0.350*
	Mental confusion	0.163*	0.092	0.366*	0.229*	0.243*	0.120	0.136	0.159*	0.385*

NPRS, Numeric Pain Rating Scale; HADS, Hospital Anxiety and Depression Scale; PCTS, Pain-Related Catastrophizing Thoughts Scale; D1, philosophic beliefs about pain; D2, denial that mood affects pain; D3, denial that pain affects mood; D4, perception of blame; D5, inadequate support; D6, disability entitlement; D7, desire for medical breakthrough; D8, skepticism of multidisciplinary approach; D9, conviction of hopelessness.

 The reliability and internal consistency were assessed using a subsample (n = 57). Adequate reliability was demonstrated for all the CRPP scale domains, with ICC values ranging from 0.786 to 0.942. The Cronbach’s alpha values were also adequate (0.714–0.922) (Table 4). No ceiling or floor effects were observed. 

**Table 4:** Reliability and internal consistency of the Cognitive Risk Profile for Pain scale (CRPP) (n = 57)

**Domain**	**Test**	**Retest**	**ICC**	**95% CI**	**SEM**	**MDC**	**α**
D1	4.44 (0.86)	4.4 (0.84)	0.856	0.756, 0.915	0.32	0.89	0.781
D2	2.72 (0.99)	2.68 (1.07)	0.856	0.755, 0.915	0.39	1.08	0.852
D3	2.93 (1.23)	2.98 (1.21)	0.93	0.882, 0.959	0.32	0.89	0.919
D4	2.83 (1.19)	2.89 (1.12)	0.87	0.779, 0.923	0.42	1.15	0.778
D5	3.17 (1.02)	3.07 (0.97)	0.898	0.827, 0.94	0.33	0.91	0.714
D6	2.52 (1.3)	2.41 (1.27)	0.907	0.842, 0.945	0.39	1.09	0.913
D7	4.28 (0.89)	4.37 (1.02)	0.882	0.8, 0.931	0.33	0.92	0.844
D8	1.75 (0.65)	1.84 (0.63)	0.786	0.637, 0.874	0.3	0.82	0.795
D9	3.54 (1.29)	3.64 (1.39)	0.942	0.901, 0.966	0.32	0.89	0.922

D1, philosophic beliefs about pain; D2, denial that mood affects pain; D3, denial that pain affects mood; D4, perception of blame; D5, inadequate support; D6, disability entitlement; D7, desire for medical breakthrough; D8, skepticism of multidisciplinary approach; D9, conviction of hopelessness; ICC, intraclass correlation coefficient; SEM, standard error of measurement; MDC, minimum detectable change; α, Cronbach’s alpha.

## DISCUSSION

 This is the first study to translate, cross-culturally adapt, and validate the CRPP scale for Brazilians with chronic pain according to international recommendations.^5-7^ Our results showed adequate construct validity, reliability, and internal consistency when applied to patients with chronic pain. 

 In terms of construct validity, the nine domains of the CRPP scale^4^ were correlated with pain,^8^ anxiety,^9^ depression,^9^ catastrophizing thoughts,^10^ tension, anger, vigor, fatigue, and mental confusion^11^ in instruments previously validated in the same population. Therefore, the CRPP scale can be used to measure philosophical beliefs about pain, denial that mood affects pain, perception of blame, inadequate support, disability entitlement, desire for medical breakthrough, skepticism of a multidisciplinary approach, and conviction of hopelessness in the assessment routine.^4^

 In terms of reliability and internal consistency, our results indicated that each of the nine domains of the CRPP scale^4^ achieved reliable scores within the errors inherent in prospective assessments. These reliability and internal consistency results allow healthcare professionals to compare the prognoses of their patients over the treatment period, as measurements at different times are supported by ICC, internal consistency, SEM, and MDC. 

 These findings have significant clinical and scientific implications. As such, the minor adjustments made to items 9 and 39 of the Brazilian version of the CRPP scale demonstrate the need for cultural adaptation beyond translation, particularly in healthcare systems with different structures and terminologies. These adaptations ensured greater clarity and relevance for the Brazilian respondents, which is crucial for the valid application of self-report instruments in diverse sociocultural contexts. 

 Additionally, the level of comprehension of 52 out of 53 items by all participants indicated a strong validity. This confirmed that the instrument is accessible to Brazilian patients with chronic pain. This is particularly relevant in clinical settings where tools must be easily understood to ensure accurate self-reporting. 

 This study has one key limitation. We only performed the analysis on a sample of Brazilian participants. To further validate the CRPP scale for chronic pain, new studies testing the CRPP scale in different languages and in different countries are warranted. Other measurement properties, such as structural validity and responsiveness, should also be considered. 

## CONCLUSION

 The Brazilian version of the CRPP scale showed adequate measurement validity in patients with chronic pain. This version of the CRPP scale can help health professionals working with Brazilians to identify maladaptive pain beliefs and coping strategies, thus enabling more personalized interventions. It can also serve as a valuable outcome measure in multidisciplinary pain management programs, helping monitor cognitive and behavioral progress over time. From a research perspective, having a culturally adapted CRPP scale allows for cross-cultural comparisons and longitudinal studies to examine how pain beliefs and behaviors evolve in response to different treatments. 

## Data Availability

The data and materials used in this study are available from the corresponding author upon request (André Pontes-Silva).
